# Comparison of Growth Kinetics of Various Pathogenic *E. coli* on Fresh Perilla Leaf

**DOI:** 10.3390/foods2030364

**Published:** 2013-08-02

**Authors:** Juhui Kim, Eunyoung Ro, Kisun Yoon

**Affiliations:** Department of Food and Nutrition, 1 Hoeki-dong, Dongdaemun-gu, Kyung Hee University, Seoul 130-701, Korea; E-Mails: wngml2316@gmail.com (J.K.); hoi-f-eun@hanmail.net (E.R.)

**Keywords:** *E. coli* O157:H7, pathogenic *E. coli*, perilla leaves, growth model, validation

## Abstract

Growth kinetics for *Escherichia coli* O157:H7 in perilla leaves were compared to those of pathogenic *E. coli* strains, including enteropathogenic (EPEC), enterotoxigenic (ETEC), enteroinvasive (EIEC) and other enterohemorrhagic (EHEC) at 13, 17, 24, 30 and 36 °C. Models for lag time (LT), specific growth rate (SGR) and maximum population density (MPD) as a function of temperature were developed. The performance of the models was quantified using the ratio method and an acceptable prediction zone method. Significant differences in SGR and LT among the strains were observed at all temperatures. Overall, the shortest LT was observed with *E. coli* O157:H7, followed by EPEC, other EHEC, EIEC and ETEC, while the fastest growth rates were noted in EPEC, followed by *E. coli* O157:H7, ETEC, other EHEC and EIEC. The models for *E. coli* O157:H7 in perilla leaves was suitable for use in making predictions for EPEC and other EHEC strains.

## 1. Introduction

Enteric *Escherichia coli* have been classified on the basis of virulence properties, including enterohemorrhagic *E. coli* (EHEC), enterotoxigenic *E. coli* (ETEC), enteropathogenic *E. coli* (EPEC), enteroadherent *E. coli* (EAEC) and enteroinvasive *E. coli* (EIEC) [1]*.* Among them, enterohemorrhagic *E. coli* O157:H7 are responsible for numerous outbreaks associated with the consumption of fresh produce in many parts of the world. From 1995 to 2006, a total of 22 outbreaks in California were directly associated with *E. coli* O157:H7 contaminated fresh lettuce or spinach [2]*.* Recently, an outbreak of the virulent strain of *E. coli* O104:H4 in vegetable sprouts grown in an organic farm has killed 35 and sickened 3256 in Germany [3]*.* From 2006 to 2012, 233 (12.5%) outbreaks in Korea were also due to pathogenic *E. coli*, out of 1871 foodborne disease outbreaks reported [4]. According to a recent epidemiological analysis regarding pathogenic *E. coli* outbreaks [5], the EPEC (44.7%) strain was reported as the primary cause of outbreak associated with *E. coli* in Korea, followed by ETEC (34.2%), EAEC (10.5%) and EHEC (9.2%). 

Consumption of fresh vegetables contaminated with *E. coli* O157:H7 poses a serious risk to humans, as epidemiological studies showed that an infectious dose may be as low as 10 cells [6]. Perilla leaf is one of the most widely consumed raw or pickled leaf vegetables in Korea and is easily contaminated with various pathogens [7]. The results of microbiological hazard analysis on fresh vegetables indicate that *E. coli* was detected in 33% to 53% of perilla leaves at the contamination level of 1.18~3.45 log CFU/g [8]. Jung *et al.* [9] compared the contamination level of *E. coli* in fresh produce in Korea and observed that the highest contamination level of *E. coli* among the tested produce was detected in perilla leaves (35.0%), which is a widely consumed fresh produce in Korea. Park *et al.* [10] also reported a higher frequency of outbreaks of foodborne illness related to perilla leaves.

Predictive models can be used to assess the impact of food handling and storage conditions on pathogen levels in food and the risk to public health [11]. Various models that predict the growth of *E. coli* O157:H7 have been developed in broth and foods [12,13,14,15,16,17]*.* Although fresh produce has been identified as a vehicle for foodborne illness caused by *E. coli* O157:H7 contamination, a few predictive models for the growth of *E. coli* O157:H7 on iceberg lettuce [18] and minimally processed leafy green vegetables have been reported [19]*.*

In the present study, a predictive model for growth of *E. coli* O157:H7 in perilla leaves was developed and then evaluated for its prediction of growth with four pathogenic *E. coli* strains, including EPEC, ETEC, EIEC and other EHEC. The purpose of this study was to determine whether or not the growth model developed with *E. coli* O157:H7 in perilla leaves can be used for growth prediction of other pathogenic *E. coli*.

## 2. Experimental Section

### 2.1. Bacterial Strains

Enteropathogenic *E. coli* (EPEC: NCCP 13715), enterotoxigenic *E. coli* (ETEC: NCCP 13717 and 13718), enteroinvasive *E. coli* (EIEC: NCCP 13719) and other enterohemorrhagic *E. coli* (EHEC: NCCP 13720 and 13721) were obtained from the Korean Food Drug Administration (KFDA) and were maintained in tryptic soy broth (TSB, Difco, Sparks, MD, USA) that contained 20% glycerol (Sigma-Aldrich, St. Louis, MO, USA) at −80 °C. Ten microliters (10 μL) of thawed stock culture was inoculated into a 50 mL Erlenmeyer flask containing 10 mL of TSB, which was then sealed [19] with a silicone cap and incubated at 36 °C for 24 h on a rotary shaker (VS-8480SR, Vision, Korea) at 140 rpm. Viable cell counts ranged from 8.5–9.5 log CFU/mL after incubation.

### 2.2. Inoculation of Strain in Perilla Leaves

Perilla leaves were washed twice with running tap water and then rinsed with distilled water for 1 min. The rinsed perilla leaves were submerged in a solution of 3.6% hydrogen peroxide for 5 min to remove background microorganisms, rinsed with distilled water and cleaned with sterilized distilled water [10]*.* The sanitized perilla leaves were air-dried in a bio-safety cabinet at room temperature for about 1 h before inoculation. The sanitized perilla leaves were then immersed for 3 min in inoculum solution (2 L), which was prepared by transferring l mL of pathogenic *E. coli* O157:H7 strains or other pathogenic *E. coli* strains into 2 L of sterile distilled water. Each sample was air-dried in a bio-safety cabinet at room temperature for 1 h, and 5 g of inoculated perilla leaves were aseptically packed into sterile bags and stored at 13, 17, 24, 30 or 36 °C. Each sample was homogenized (Stomacher, Interscience, Paris, France) for 2 min in 40 mL of 0.1% sterilized peptone water. One milliliter (1 mL) of homogenized sample was diluted in 9 mL of 0.1% sterilized peptone water. One hundred microliter (100 μL) aliquots of two dilutions of each sample were plated on eosin methylene blue agar (EMB: Difco, Sparks, MD, USA) in duplicate and incubated aerobically at 36 °C for 24 h. The colonies on duplicate plates of each sample were counted and, then, converted to log numbers. Experiments were replicated twice for each strain and storage temperature. 

### 2.3. Primary Modeling

Viable counts (log CFU per g) of *E. coli* O157:H7 and other pathogenic *E. coli* were graphed as a function of time and, then, iteratively fitted to the modified Gompertz model using GraphPad PRISM V4.0 (GraphPad Software, San Diego, CA, USA) to determine the lag time (LT), specific growth rate (SGR) and maximum population density (MPD). The model used was as follows:

*Y*_0_ = *N*_0_ + *C* × exp{−exp[(2.718 × SGR/*C*) × (LT − *t*) + 1]} (1)

where *Y*_0_ is the viable cell count (log CFU per g) at time *t* (h), *N*_0_ is the initial log number of cells, *C* is the difference between the initial and final cell numbers, SGR is the maximum specific growth rate (log CFU per h), LT is the lag time before growth and *t* is the incubation time. The goodness-of-fit of the data was evaluated based on the coefficient of determination (*R*^2^), which was provided by GraphPad PRISM.

### 2.4. Secondary Modeling

LT, SGR and MPD values were graphed as a function of temperature and then fitted to the Davey, square root and polynomial models, respectively. The Davey model used was as follows [20,21]:

*Y* = *a* + (*b*/*T*) + (*c*/*T*^2^) (2)

where *Y* is LT (h), *a*, *b* and *c* are regression coefficients without biological meaning and *T* is temperature.

The square-root model used was as follows [22] : 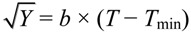
(3)
where *Y* is SGR (log CFU/ h), *b* is a regression coefficient, *T* is temperature (°C) and *T*_min_ is the cardinal minimum growth temperature.

The second order polynomial model used was as follows [23]*:*

*Y* = *a* + *b* × *T* + *c* × *T*^2^ (4)

where *Y* is MPD (log CFU) and *a*, *b* and *c* are regression coefficients without biological meaning.

### 2.5. Performance Evaluation of Perilla Leaves Model

Different pathogenic *E. coli* strains, including EPEC, ETEC, EIEC and other EHEC, were used for performance evaluation of *E. coli* O157:H7 models for perilla leaves. The performance of the models was quantified using the ratio method described by Ross [24] and an acceptable prediction zone method [25]. Prediction bias (*B_f_*) and accuracy (*A_f_*) factors were calculated using the following equations [26]:

*B_f_* for LT = 10^∑log(predicted/observed)/*n*^ (5)

*A_f_* for LT = 10^∑|log(predicted/observed)|/*n*^ (6)

*B_f_* for SGR = 10^∑log(observed/predicted)/*n*^ (7)

*A_f_* for SGR = 10^∑|log(observed/predicted)|/*n*^ (8)

where *n* is the number of prediction cases used in the calculation. Different ratios were used for LT and SGR, so that *B_f_* less than 1 would represent fail-safe predictions and *B_f_* and *A_f_* above 1 would represent fail-dangerous predictions [26]. In addition, relative errors (RE) of individual prediction cases were calculated using the following equations [27]:

RE for LT (%) = [(predicted − observed)/predicted] × 100
(9)

RE for SGR or MPD (%) = [(observed − predicted)/predicted] × 100
(10)

where RE less than zero represented fail-safe predictions and RE above zero represented fail-dangerous predictions. The median relative error (MRE) and the mean absolute relative error (MARE) were also used to measure the prediction bias and accuracy of the model, respectively. 

In the acceptable prediction zone method for LT, SGR and MPD, the percentage of RE (%RE) that is in an acceptable prediction zone (*i.e.*, the ratio of the number of RE in the acceptable prediction zone to the total number of prediction cases) from −30% (fail-safe) to 15% (fail-dangerous) was calculated and used also as a measure of model performance [25,26].

### 2.6. Statistical Analysis

The values of LT, SGR and MPD were also analyzed by analysis of variance, and the means were separated using Duncan’s multiple range test at *p* < 0.05 using the Statistical Analysis Systems (SAS) V 9.1 (SAS Institute Inc., Cary, NC, USA).

## 3. Results and Discussion

### 3.1. Development of Growth Model for *E. coli* O157:H7 in Perilla Leaves

Perilla leaves was used as a substrate to develop a growth model of *E. coli* O157:H7 (NCTC 12079) as a function of time and temperature. Although the growth of *E. coli* O157:H7 in TSB was observed at 7 and 10 °C (data not shown), the growth of *E. coli* O157:H7 in perilla leaves was not observed under 12 °C. Therefore, growth kinetics for *E. coli* O157:H7 in perilla leaves was compared at 13, 17, 24, 30 and 36 °C. Especially, the MPD of *E. coli* O157:H7 in perilla leaves was significantly decreased at 13 and 17 °C. The LT, SGR and MPD secondary models for perilla leaves as a function of temperature were also developed using the Davey, square-root and polynomial models, respectively (Table 1). Park *et al.* [10] observed growth of *L. monocytogenes* in perilla leaves at only 24 °C, and LT and SGR of *L. monocytogenes* in perilla leaves was 7.92 h and 0.028 log CFU/h, respectively. On the other hand, shorter LT (5.52 h) and faster SGR (0.207 log CFU/h) were observed at 24 °C with *E. coli* O157:H7 in the current study. This indicates that the growth of *E. coli* O157:H7 in perilla leaves is faster than that of *L. monocytogenes* at 24 °C.

**Table 1:** Lag time, SGR and MPD of *E. coli* O157:H7 in perilla leaves and developed secondary models.

Parameter	13 °C		17 °C		24 °C		30 °C		36 °C		Secondary model equation
	Mean	SE	Mean	SE	Mean	SE	Mean	SE	Mean	SE	
LT ^x^	45.84 ^e^	0.16	20.40 ^d^	0.23	4.80 ^c^	0.10	2.64 ^b^	0.10	2.40 ^a^	0.32	LT = 0.6688 + (−42.71/*T*) + (763.7/*T*^2^)
SGR ^y^	0.034 ^a^	0.00	0.055 ^b^	0.01	0.236 ^c^	0.02	0.359 ^d^	0.05	0.548 ^e^	0.00	SGR = [0.1183(*T* − 5.182)]^2^
MPD ^z^	5.73 ^a^	0.11	5.81 ^a^	0.10	6.73 ^b^	0.12	7.01 ^b^	0.03	7.02 ^b^	0.04	MPD = 3.717 + 0.1769*T* − 0.002378*T*^2^

^x^ LT, lag time (h); ^y^ SGR, specific growth rate (log CFU/h); ^z^ MPD, maximum population density (log); ^a–e^ Mean values (*n* = 4) in the row followed by different letters are significantly different (*p* <0.05); *T*, temperature.

### 3.2. Evaluation of Model Performance

The performance of the growth model can be evaluated for the dependent data used during model development and for independent data not used during model development. In the present study, the growth model of *E. coli* O157:H7 in perilla leaves was evaluated using independent sets of data obtained with EPEC (enteropathogenic *E. coli* NCCP 13715), a mixture of ETEC (enterotoxigenic *E. coli* NCCP 13717 and 13718), EIEC (enteroinvasive *E. coli* NCCP 13719) and a mixture of other EHEC (enterohemorrhagic *E. coli* NCCP 13720 and 13721), which are other strains than the ones used in the model development. Table 2 shows that predicted SGR and LT values by secondary models for *E. coli* O157:H7 in perilla leaves were compared to the observed SGR and LT values with 4 different strains (EPEC, ETEC, EIEC and other EHEC). Significant differences in SGR and LT values in perilla leaves were observed, depending on the type of strains at all tested temperatures (*p* < 0.05). The differences in LT values were increased between the predicted data with the *E. coli* O157:H7 model and the observed data with various pathogenic *E. coli* strains at lower storage temperature. At 13 °C, the shortest LT was observed with *E. coli* O157:H7 in perilla leaves, followed by EPEC, EHEC, other, EIEC and ETEC, while the fastest growth rates were noted in EPEC, followed by *E. coli* O157:H7 in perilla leaves at 13 °C.

**Table 2:** Comparison of growth kinetics * for *E. coli* O157:H7 to those ** for various pathogenic *E. coli* strains in perilla leaves.

Parameter	Strains	13 °C		17 °C		24 °C		30 °C		36 °C	
		Mean	SE	Mean	SE	Mean	SE	Mean	SE	Mean	SE
LT ^x^	O157:H7	45.84 ^a^	0.00	19.68 ^d^	0.00	5.52 ^b^	0.00	2.64 ^a^	0.00	2.16 ^a^	0.00
	EPEC ^k^	47.52 ^b^	1.18	19.44 ^c^	1.76	6.48 ^b^	0.43	2.73 ^a^	0.06	2.64 ^b^	0.01
	ETEC ^l^	78.48 ^e^	0.85	11.52 ^a^	0.29	3.60 ^a^	0.30	2.89 ^b^	0.20	2.88 ^c^	0.07
	EIEC ^m^	55.92 ^c^	1.06	23.28 ^e^	0.55	7.68 ^c^	0.01	2.74 ^a^	0.11	2.87 ^c^	0.04
	EHEC ^n^	54.24 ^d^	1.42	18.48 ^b^	0.24	5.04 ^b^	0.05	2.88 ^b^	0.07	2.12 ^a^	0.04
SGR ^y^	O157:H7	0.036 ^d^	0.00	0.081 ^b^	0.00	0.207 ^e^	0.00	0.359 ^d^	0.00	0.554 ^c^	0.00
	EPEC	0.040 ^e^	0.01	0.084 ^c^	0.00	0.204 ^d^	0.01	0.347 ^c^	0.02	0.555 ^d^	0.07
	ETEC	0.027 ^c^	0.00	0.107 ^d^	0.01	0.161 ^a^	0.00	0.332 ^b^	0.01	0.455 ^a^	0.04
	EIEC	0.015 ^a^	0.00	0.079 ^a^	0.09	0.192 ^c^	0.01	0.298 ^a^	0.01	0.555 ^d^	0.00
	EHEC	0.026 ^b^	0.02	0.084 ^c^	0.00	0.182 ^b^	0.04	0.396 ^e^	0.02	0.499 ^b^	0.02

* Predicted value (*n* = 4) from the secondary model for *E. coli* O157:H7 on perilla leaves; ** Observed value (*n* = 4); ^a–e^ Mean values (*n* = 4) in the column followed by different letters are significantly different (*p* < 0.05); ^x^ LT, lag time (h); ^y^ SGR, specific growth rate (log CFU/h); ^k^ EPEC, enteropathogenic; ^l^ ETEC, enterotoxigenic; ^m^ EIEC, enteroinvasive; ^n^ EHEC, enterohemorrhagic.

**Figure 1:**
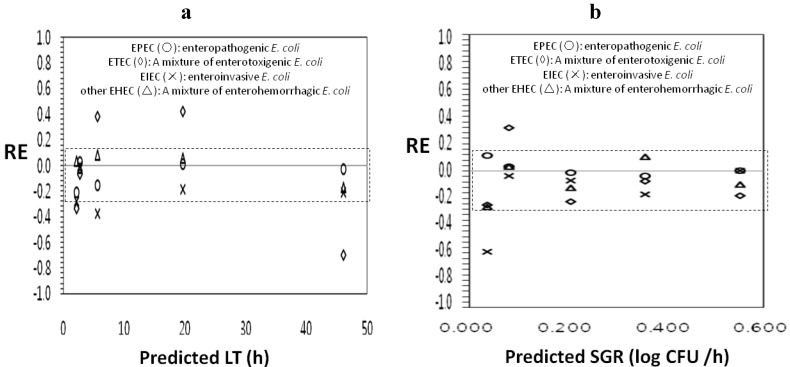
Relative error (RE) plots with an acceptable prediction zone of lag time (LT) and specific growth rate (SGR) data used for model performance of extrapolation. (**a**) LT; (**b**) SGR.

Table 3 shows the performance of secondary growth models for *E. coli* O157:H7 in perilla leaves for prediction of other pathogenic *E. coli* strains. In the LT model for perilla leaves, EIEC had the lowest *B_f_* of 0.83, indicating that the model developed for *E. coli* O157:H7 in perilla leaves predicted the LT that was 17% shorter than those actually observed for EIEC in perilla leaves. All %RE of EIEC were less than zero (Figure 1), which indicated fail-safe predictions [26], and three of five REs (percentage of RE = 66.7%) for the EIEC model were inside the acceptable prediction zone. However, ETEC had a *B_f_* of 1.03, indicating that the growth model of *E. coli* O157:H7 in perilla leaves predicted LT that was 3% slower than those actually observed for ETEC in perilla leaves, and only one of five relative errors (percentage of RE = 20%) for these models were inside the acceptable prediction zone. These results indicate that the LT model in perilla leaves for *E. coli* O157:H7 was not suitable for ETEC and EIEC. On the other hand, *B_f_* for the LT model to EPEC and other EHEC strains was acceptable, at values of 0.94 and 1.00, respectively, and the RE plot also indicated that all REs (100%) for LT were inside the acceptable prediction zone, indicating that the LT model in perilla leaves for *E. coli* O157:H7 was suitable for EPEC and other EHEC strains.

In the SGR model, EIEC had the lowest *B_f_* of 0.79, indicating that the growth model of *E. coli* O157:H7 in perilla leaves predicted the SGR that was 21% faster than those actually observed for EIEC in perilla leaves (Table 3). All REs of EIEC were less than zero, which indicated fail-safe predictions, and four of five relative errors (percentage of RE = 80%) for these models were inside the acceptable prediction zone. However, EPEC had the highest *B_f_* of 1.02 Moreover, the *B_f_* of ETEC and other EHEC were acceptable, 0.90 and 0.92, respectively, indicating that the model predicted an SGR that was 10% and 8% faster than those actually observed for ETEC and other EHEC in perilla leaves, respectively. All REs (percentage of RE = 100%) for EPEC and other EHEC were inside the acceptable prediction zone. These results show that LT and SGR secondary models of perilla leaves for *E. coli* O157:H7 were suitable for enteropathogenic *E. coli* (EPEC) and other enterohemorrhagic *E. coli* (EHEC), but only the SGR model of *E. coli* O157:H7 predicted for the growth of enteroinvasive *E. coli* (EIEC) and enterotoxigenic *E. coli* (ETEC) in perilla leaves well. 

**Table 3:** Performance of secondary growth models for *E. coli* O157:H7 in perilla leaves for various pathogenic *E. coli* strains.

Strain	Model	*B_f_* ^a^	MRE ^b^	*A_f_* ^c^	MARE ^d^	%RE ^e^
EPEC ^f^	LT ^j^	0.94	−0.03	1.08	0.09	100
	SGR ^k^	1.02	0.14	1.04	0.04	100
ETEC ^g^	LT	1.03	−0.07	1.46	0.38	33.3
	SGR	0.90	−17.92	1.24	0.21	83.3
EIEC ^h^	LT	0.83	−0.21	1.21	0.21	66.7
	SGR	0.79	−7.20	1.27	0.17	83.3
EHEC ^i^	LT	1.00	0.04	1.08	0.08	100
	SGR	0.92	−9.87	1.14	0.12	100

^a^ *B_f_*, bias factor; ^b^ MRE, median relative error; ^c^ *A_f_*, accuracy factor; ^d^ MARE, mean absolute relative error; ^e^ %RE, the percentage of relative error in an acceptable prediction range from −30% to 15% for SGR and −60% to 30% for LT; ^f^ EPEC, enteropathogenic; ^j^ LT, lag time (h); ^k^ SGR, specific growth rate (log CFU/h); ^g^ ETEC, enterotoxigenic; ^h^ EIEC, enteroinvasive; ^i^ EHEC, other enterohemorrhagic *E. coli*.

A well-known strategy for modeling is to choose the fastest growing strain in the environmental conditions investigated, because the fastest growing strain will dominate the growth in food products [28]. McMeekin *et al.* [23] also recommended independent modeling of several different strains before choosing the strain that grows fastest under the environment conditions of most interest. Salter *et al.* [29] compared the growth of the nonpathogenic, *E. coli* M23, with the growth of pathogenic strains of *E. coli* O157:H7 and found only small differences in the growth responses of these two strains. They also found that the model based on *E. coli* M23 was able to describe the growth of pathogenic strains of *E. coli* O157:H7.

## 4. Conclusions

Developed LT and SGR models for *E. coli* O157:H7 in perilla leaves were suitable for only enteropathogenic *E. coli* (EPEC) and other enterohemorrhagic *E. coli* (EHEC). As a result of comparison of growth kinetics in perilla leaves, *E. coli* O157:H7 and EPEC are the high risk ones of pathogenic *E. coli* among the pathogenic *E. coli*. The results of the current study provide the growth characteristics of various pathogenic *E. coli* strains in perilla leaves at various temperatures, which will be useful information for risk assessment of pathogenic *E. coli* in perilla leaves at a retail fresh market.
